# Testing the feasibility of the startle-first route to deimatism

**DOI:** 10.1038/s41598-018-28565-w

**Published:** 2018-07-16

**Authors:** Grace G. Holmes, Emeline Delferrière, Candy Rowe, Jolyon Troscianko, John Skelhorn

**Affiliations:** 1 Centre for Behaviour & Evolution, Institute of Neuroscience, Newcastle University, Newcastle upon Tyne, UK; 2 Centre for Ecology and Conservation, College of Life & Environmental Sciences, University of Exeter, Exeter, UK

## Abstract

Many prey species perform deimatic displays that are thought to scare or startle would-be predators, or elicit other reflexive responses that lead to attacks being delayed or abandoned. The form of these displays differs among species, but often includes prey revealing previously-hidden conspicuous visual components. The evolutionary route(s) to deimatism are poorly understood, but it has recently been suggested that the behavioural component of the displays evolves first followed by a conspicuous visual component. This is known as the “startle-first hypothesis”. Here we use an experimental system in which naïve domestic chicks forage for artificial deimatic prey to test the two key predictions of this hypothesis: (1) that movement can deter predators in the absence of conspicuously coloured display components; and, (2) that the combination of movement and conspicuously coloured display components is more effective than movement alone. We show that both these predictions hold, but only when the movement is fast. We thus provide evidence for the feasibility of ‘the startle-first hypothesis’ of the evolution of deimatism.

## Introduction

The study of animal colouration continues to be an important testbed for evolutionary theory, and has played a key role in enhancing our understanding of a number of areas of biology^1^. Perhaps one of the most well-studied functions of animal colouration is its role in defending prey from predators^2–10^, yet there are several forms of defensive colouration that remain poorly understood. Deimatic displays, for example, have long been recognised as a discrete form of defence^11–17^, and are thought to scare or startle predators^6,13,14,18^, or trigger other reflexive responses that cause predators to delay or abandon their attacks^19^. These displays occur across a wide range of different animal groups, [well-known examples are found in cephalopods^20^, insects^12,21–25^, and amphibia^26^], and often involve prey adopting characteristic movements and postures while revealing conspicuous visual display components to an attacking predator. For example, cuttlefish (*Sepia officinalis*) flatten their bodies, extend their peripheral fin and suddenly change colour to present a conspicuous dark eyespot pattern on the rear of the mantle and a dark contour that runs parallel to the body’s edge^20^; and underwing moths (*Catocala* spp.) open their cryptic forewings to reveal striking, conspicuously patterned hindwings^16,27^. There is some evidence that this kind of display can deter predators^22,23,25,28^, however, we know very little about the evolutionary pathways via which these displays evolve^29^.

It is unlikely that visual and behavioural components of deimatic displays evolved simultaneously. This raises the question of whether the conspicuous colours and patterns often associated with deimatic displays evolve before or after the behavioural component of the displays (i.e. the characteristic movements and/or postures)^19^? Since hidden visual display components are likely to be useless without the movement that reveals them, Umbers *et al*. (2017) recently suggested that in undefended prey at least, the behavioural component of the display evolves first followed by the conspicuous colours and patterns^19^ (see^19^ for other potential routes to deimatism). They refer to this as ‘the startle-first hypothesis’^19^, and it relies on two key assumptions: that the behavioural components of deimatic displays have a deterrent effect on predators, and that this is enhanced by the evolution of conspicuous visual display components. However, these assumptions have not been tested.

Here, we critically test the predictions that: (1) movement alone in the absence of a conspicuous visual component is sufficient to deter predators; and, (2) the combined effect of movement and a conspicuous visual component is more effective than movement alone. We used an experimental system in which naïve domestic chicks (*Gallus gallus domesticus*) attacked computer-generated moth-like deimatic prey projected onto the floor of an experimental arena, in order to test these predictions. After learning to attack artificial prey, each chick received one test trial in which it was presented with a single prey item. They type of prey chicks received differed among our seven experimental groups. Chicks received either Control (stationary background-matching prey), Deimatic (opened their forewings to reveal conspicuous red hindwings) or, Background-matching prey (opened their forewings to reveal background-matching hindwings). Deimatic and Background-matching prey produced their displays at one of three different speeds (Slow, Moderate, or Fast). This allowed us to determine whether or not movement speed could influence the benefits to displaying and consequently, the likelihood that deimatic displays would be selected for.

## Results

**Figure 1:**
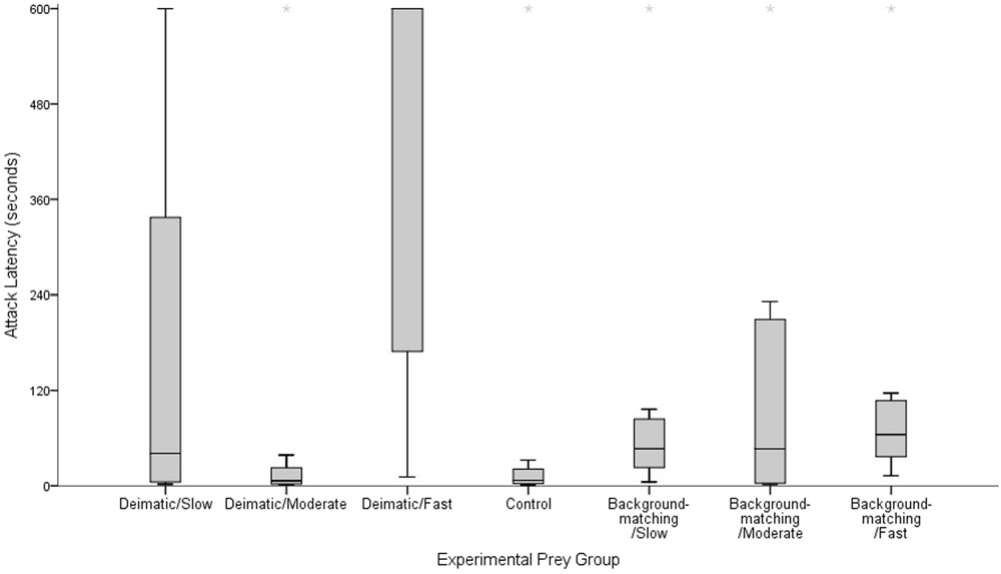
Latency to attack prey during the test trial. Each box represents the middle 50% of scores for each experimental group. The line dividing each box into two represent the median of those data. The upper and lower whiskers extend to include the data outside the middle 50%. Asterisks (*) represent possible outliers, each of which has been examined and is valid for inclusion within the analysis.

We found that the time that chicks took to attack prey differed among our experimental groups (Kruskal-Wallis test: χ^2^ = 16.571, *p* = 0.011, df = 6; Fig. 1). As predicted (see methods and materials section), chicks in the Background-matching/Fast group took significantly longer to attack prey than chicks in the Control group (Kruskal-Wallis test: χ^2^ = 6.1, *p* = 0.014, *df* = 1). Contrary to our predictions, there was no significant difference in attack latency between the Background-matching/Moderate group and the Control group (Kruskal-Wallis test: χ^2^ = 0.798, *p* = 0.372, *df* = 1), or between the Background-matching/Slow group and the Control group (Kruskal-Wallis test: χ^2^ = 3.780, *p* = 0.052, *df* = 1). However, the latter approached significance and so should be interpreted with caution. These results indicate that, fast movement can be an effective deterrent in the absence of other conspicuous visual display components.

As predicted, chicks in the Deimatic/Fast group took significantly longer to attack prey than chicks in the Background-matching/Fast group (Kruskal-Wallis test: χ^2^ = 4.422, *p* = 0.033, *df* = 1), attacking the Deimatic/Fast (M = 418.69 s, SE = 90.14 s) prey over three times faster than Background-matching/Fast (M = 128.68 s, SE = 68.47 s) prey. However, we found no significant difference in the attack latencies between chicks in the Deimatic/Moderate group and chicks in the Background-matching/Moderate group (Kruskal-Wallis test: χ^2^ = 1.461, *p* = 0.246, *df* = 1), or between chicks in the Deimatic/Slow group and chicks in the Background-matching/Slow group (Kruskal-Wallis test: χ^2^ = 0.100, *p* = 0.805, *df* = 1). This indicates that conspicuous colouration enhanced the deterrent effect of movement, but only when prey moved at the fast speed.

## Discussion

Many deimatic displays involve prey revealing previously-hidden conspicuous visual components when approached or attacked by a predator^11,14,25,27^. Understanding how these displays evolved has proved challenging because the conspicuous visual components are unlikely to be effective without the movement that reveals them, and it is unlikely that the visual components and the behaviour evolved simultaneously. Our results provide clear support for the two key predictions underlying the startle-first hypothesis, whereby movement is thought to evolve before conspicuous display components. We found that fast movement by prey was sufficient to cause birds to delay their attacks, and the combined effect of fast movement and a novel conspicuous visual component was more effective than movement alone. It is therefore feasible that natural selection could initially favour displays in which prey quickly move body parts, and could subsequently favour the evolution of conspicuous visual components that augment these behavioural displays^19^.

Our findings also suggest that deimatic displays need not necessarily include conspicuous components, other than fast movement, in order to be effective. This suggests that deimatism could evolve even in situations where there are factors constraining the evolution of other conspicuous display components, as suggested in^13^. For example, where the cost of producing bright pigmentation or loud sounds is prohibitively high or restricted by an animal’s physiology^30–34^. Indeed, most studies investigating the antipredator benefits of deimatism focus on species with displays containing highly conspicuous visual and/or auditory components (e.g.^14,16,22,23,25,28,35–38^; although see^39^). This may be because most deimatic displays have them, but an alternative explanation is that we have not fully considered the possibility that species could benefit from deimatism without them. Deimatism could be more widespread than previously thought, and the form of these displays could be more diverse than we currently appreciate. For example, several cryptic species of moth (e.g. the Early Thorn moth *Selenia dentaria* and the Hebrew Character *Orthosia gothica*) occasionally flick their wings when threatened (Skelhorn, *pers. Obs*.), a movement which may be involved with preparation for flight. However, it is not difficult to imagine such a movement proving the rudimentary basis for the wing movements seen in lepidopteran displays^40,41^.

Intriguingly, we only found a significant deterrent effect of movement when the prey moved at the fastest of the three speeds used. This suggests that the speed or number of displays (since here, as in natural systems, the two are correlated) needs to exceed a particular threshold in order to have a deterrent effect. This would be consistent with (although not a critical test of) the idea that deimatic displays work by startling predators, as rapid onset (or rise time) of a startling stimulus is required in order to successfully elicit a startle response^42^. However, it is worth noting that the difference in reaction times between chicks in the Control group and chicks in the Background-matching/Slow group approached significance. We would therefore not rule out the possibility that a wider range of movement speeds could have deterrent effects on predators, and would recommend further research in this area.

It is also worth noting that our experiment used naïve predators searching for artificial deimatic prey. This was crucial to control movement speed and ensure that predators’ responses were not influenced by their previous experience with deimatic prey. However, our experiment should be considered as an initial step toward testing the order in which the various components of deimatic displays may evolve. Here we have shown that movement alone can be effective in deterring predators. The next logical step is to determine whether the movements used in real deimatic displays are effective in deterring the natural predators of deimatic species and enhancing prey survival, in the absence of other display components. The only way to truly examine such movements would be to present predators with live prey, ideally within a natural setting.

Although we have shown that this evolutionary pathway is feasible, it does not preclude the existence of other pathways. Conspicuous visual patterns have evolved in some lepidopteran species to aid in mate recognition^43^ and, as markers of individual quality in relation to sexual selection and mate choice^44^. It is possible that these existing visual patterns were incorporated into deimatic displays after the evolution of the moving and/or behavioural components. In addition, selection may favour the addition of other display components (e.g. producing a loud auditory stimulus, incorporating a movement which appears to lead to a sudden increase in size^19^) if they confer additional benefits in terms of enhancing the deterrent effect of the display or preventing habituation. It is therefore possible that there are multiple pathways to the evolution of deimatic displays.

In conclusion, we have provided evidence that the startle-first hypothesis for the evolution of deimatism is feasible, i.e. that selection could favour the evolution of the behavioural components of deimatic display followed by the evolution of other conspicuous visual features. This is the first evidence of any evolutionary pathway in relation to deimatism, and this helps us to understand how the numerous often conspicuous components came to form a cohesive defensive mechanism. It should be noted that the route outlined here relates only to those species that use movement to reveal previous hidden visual components. Some deimatic species appear to perform displays that rely on other sensory modalities^35,39^ or utilise visual components that are on constant display^45^, and further work is needed to determine how such displays evolve. As is becoming apparent with all aspects of deimatism, it is important that we do not take a “one-size-fits-all” approach, but remain aware that just as displays of different species differ, so too will their evolutionary route.

## Methods and Materials

### Subjects

Eighty newly-hatched domestic chicks (*Gallus gallus domesticus*) of the ‘Ross’ strain were used in this experiment: 56 served as experimental chicks and 24 acted as buddy chicks (see later). The chicks were of mixed sex, and were purchased in two cohorts (39 in the first cohort and 41 in the second) from a commercial hatchery in Yorkshire (U.K.) as day-old hatchlings. They were housed in a floor pen measuring 56 × 85.5 × 208 cm. The floor of the pen was covered with wood chips and contained a food hopper, a water hopper, and a plastic shelter (41 × 37 × 54.5 cm). The birds were subject to a 12:12 hour light:dark cycle beneath fluorescent lighting, and the laboratory was maintained at 25–29 °C using three room heaters. Water and chick starter crumbs were available *ad libitum*, except prior to training and experimentation when brief periods of food (but not water) deprivation were required to ensure the chicks’ motivation to forage.

The chicks were marked with non-toxic marker pens in order to enable individual identification, and were weighed and visually inspected daily. All of the chicks gained weight throughout the experiment, and were rehomed to a free-range farm when the experiment concluded. The experimental protocol was approved by the Newcastle University Animal Welfare and Ethical Review Board (Project ID No. 500), and followed UK Home Office Guidelines and the Association for the Study of Animal Behaviour Guidelines for the Treatment of Animals in Research and Teaching.

### Experimental arena

**Figure 2:**
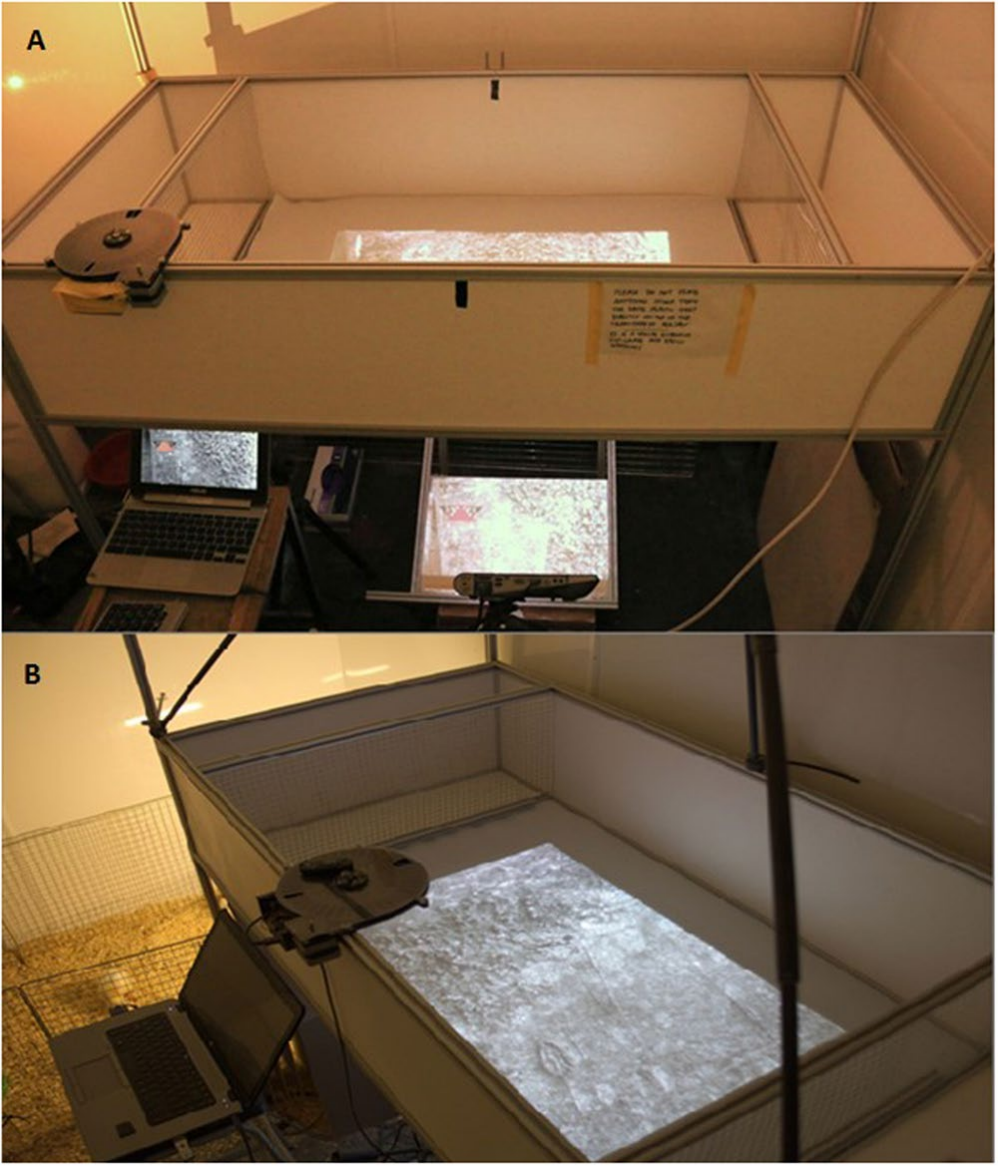
Images of arena showing the projector (**A**) and the layout of the experimental arena in the centre and buddy areas on either side, each containing two buddy chicks (**B**).

The apparatus consisted of an open-topped experimental arena (30 × 71 × 106 cm) with opaque plastic walls (see Fig. 2). At each of the narrow ends of the arena, a section measuring 30 × 30 × 71 cm was partitioned off using wire mesh, in order to create two separate ‘buddy arenas’. Each buddy arena housed two buddy chicks during all training and test trials. The presence of the buddy chicks prevented experimental chicks from becoming distressed when foraging alone in the arena. These buddy chicks were selected from a stock of 24 individuals (12 in each cohort), and were changed every six trials. Buddy chicks were not given access to artificial prey at any point during the experiment.

Images of natural backgrounds (see background image section) containing artificial prey were projected onto the arena floor from below. An Optoma ML1500 LED DLP projector positioned beneath the arena projected the images onto a mirror, which in turn reflected them onto the arena floor. The floor of the arena was made of a 0.5 mm thick sheet of white Polytetrafluoroethylene (PTFE) placed on top of a transparent 5 mm thick sheet of Polymethyl methacrylate (PMMA or acrylic glass). This structure ensured that the display looked the same to the chicks regardless of their viewing angle, an important consideration as they would be walking across the floor and viewing the projected images from various viewpoints.

### Background images

Background images were created from 57 monochromatic photographs of tree bark (oak, beech, birch, holly and ash) taken using a Canon 5D MKII with a Nikkor EL 80 mm lens at F/22 to ensure a sizable depth of field. These photographs were taken under diffuse light conditions and the images were uploaded to a computer where they were standardized to ensure they had a similar mean luminance and contrast (variance in luminance). See the supplementary material for image processing methods.

### Artificial prey

Artificial prey were loosely based on deimatic lepidoptera that repeatedly open and close their cryptic forewings when disturbed in order to flash conspicuous hindwings (e.g. Underwing moths; *Catocala* sp. and Peacock butterflies; *Aglais io*). They were not, however, intended to represent any particular species. Each prey image was triangular in shape and was generated from the background against which it was presented using code written specifically for that purpose (similar to that used in^46,47^).

### Training prey

Chicks were trained to find stationary, cryptic, triangular prey (100 × 200 pixels, or 7.4 × 3.7 cm H × W) during their first 8 days in the laboratory (see below for training protocol). Two prey types were used: background-matching prey and distractive prey^46^ with small conspicuous markings that are thought to distract attention from salient prey outlines^48^. See the supplementary material for details of how prey were produced. The use of two types of training prey was related to the chicks’ participation in an experiment investigating the effect of distractive markings on camouflage (See supplementary material for further details). All chicks participated in the camouflage experiment for the two days between the training and testing phases of this study (see below). The camouflage experiment involved two trials similar to the trials used in the training phase of this experiment, but with small variations in the number and order of background matching and distractive prey. We ensured that chicks’ experiences in the camouflage experiment were counterbalanced across the experimental groups used in this study in order to ensure that taking part in the camouflage study did not affect the outcome of this experiment (see supplementary material).

### Experimental prey

Experimental prey consisted of a background-matching triangle as above, however, these were adjusted to be symmetrical. The prey background consisted of dark pixels that covered 80% of the prey, while the pattern elements of the prey were made up of light pixels that covered the remaining 20% of the prey image (rather than 50:50 as in training). Consequently, the experimental prey were slightly easier to find than training prey, ensuring that that they were distinct from training prey, and that the chicks did not overlook them in the test trials.

**Figure 3:**
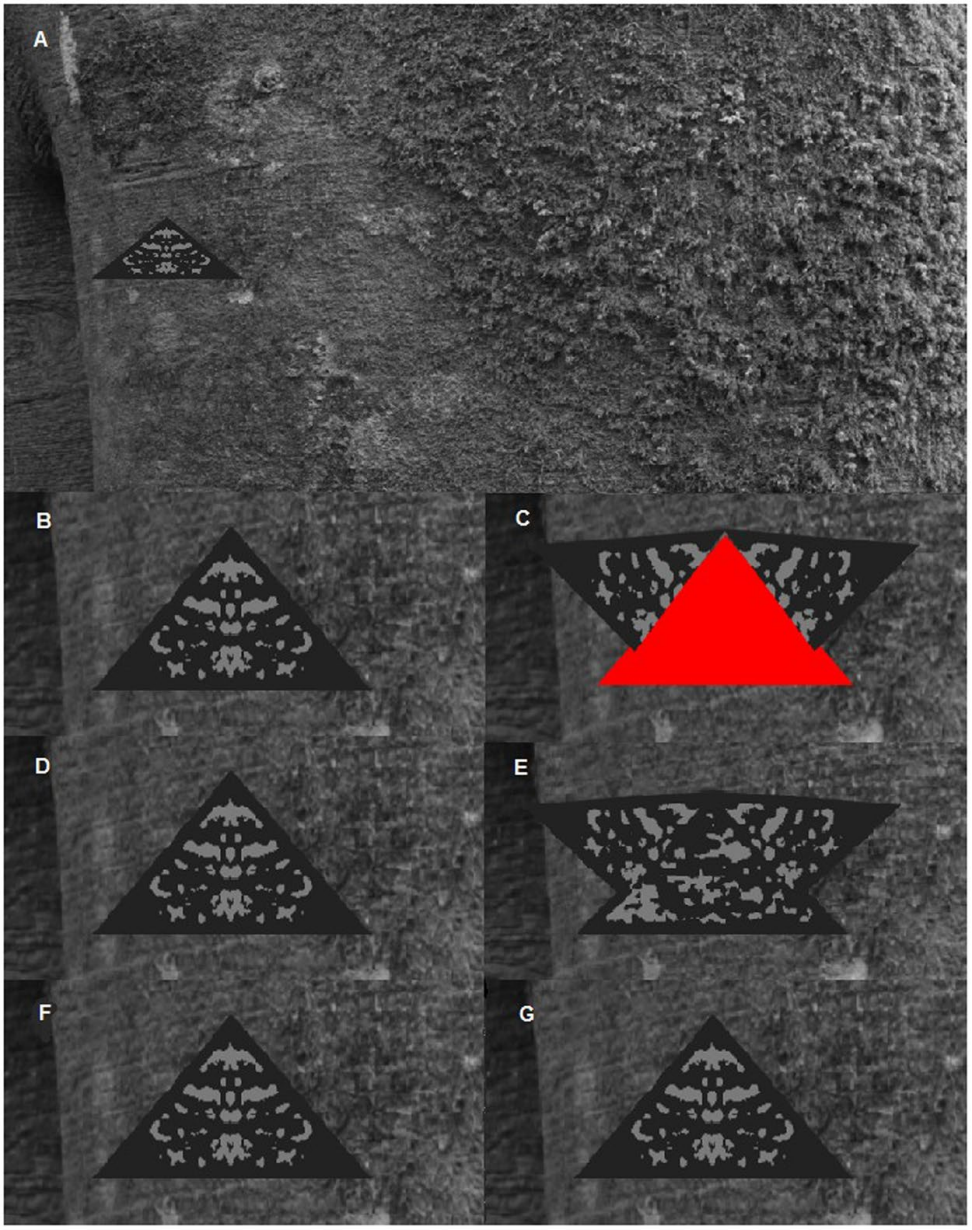
(**A**) Example of an artificial prey item on the natural bark background image. Conspicuous deimatic prey with the forewings closed (**B**) and open (**C**), revealing bright red underwing colouration. Background matching prey with the forewings closed (**D**) and open (**E**), revealing background matching underwing colouration. Control prey (**F**) with the forewings always closed (**G**).

We created static prey and six types of moving prey. When activated, the ‘forewings’ of the moving prey repeatedly opened and closed, as if hinged at the peak of the triangle, to reveal the ‘hindwings’ (see Fig. 3B). The wings flicked at one of three different speeds which fall within the range observed in live lepidoptera^23,28^: Slow, 1 flick per second (fps); Moderate, 1.8 fps; and Fast: 2.22 fps. Wing flicking revealed hindwings that were either identical to the forewings (Background-matching) or a uniform bright red colour (Deimatic), and continued from activation to the end of the trial.

### Training

On day one post-hatch, all of the chicks were placed into the experimental arena simultaneously. They were allowed to forage for mealworms (*Tenebrio molitor*) that had been scattered across the arena floor, for a period of 30 minutes. A uniformly grey slide was projected onto the arena floor during this training session. This habituated the chicks to the arena, and encouraged them to forage in subsequent trials.

From day 2 post-hatch, each of the experimental chicks received one training trial per day over eight consecutive days. Each trial involved the presentation of 20 slides, each composed of a background image containing a randomly-positioned artificial prey item. In each presentation, the background image was selected at random from a stock of 57 images, and the prey image was unique. The 20 slides in each training trial were divided into 10 groups of two slides randomly assigned as either distractive or background-matching training prey. Thus each chick received equal numbers of each prey type presented in pseudo-random order.

During initial training trials (days 2–3 post hatch), prey images were presented against a uniformly grey background. Chicks were rewarded with mealworms by the experimenter for approaching, pecking or scratching the prey image. When chicks were consistently pecking and/or scratching the prey image, we switched from the grey background, to the background images. This typically took one training trial but could take up to three training trials to achieve. We began with images at 10% opacity (so prey could be found easily), and increased the level of opacity each time the chicks successfully pecked or scratched prey without prompt multiple times, until the level of opacity reached 100%. This made the prey image more cryptic against the background across successive trials. A successful attack of the prey image was defined as pecking or scratching the prey image itself. We considered chicks to be fully trained when they successfully attacked eight prey within ten consecutive presentations on a background of between 90–100% opacity. It usually took between six and eight training trials to reach criterion. During the course of training, the delivery of the mealworm reward progressed from being distributed by the experimenter, to delivery by an automated hopper positioned on one side of the arena. This allowed us to minimise the involvement of the experimenter in the chicks’ experiences.

### Test trial

On day 12 post hatch, the fifty-six experimental chicks were randomly assigned to one of seven experimental groups (N = 8 for each group), with the caveat that chicks’ experiences on days 10 and 11 were counterbalanced across groups in order to minimise any possible effect of the experimental treatments in the camouflage experiment. Chicks then received a single test trial in which they were presented with a single artificial prey of one treatment type, thus providing us with the behavioural response of a completely naïve subject. There were three types of prey; Control, Background-matching and Deimatic. Control prey were stationary and background-matching. Background-matching and Deimatic prey moved their forewings either at Fast, Moderate, or Slow speeds (see supplementary video files). Background-matching prey revealed background-matching hindwings and Deimatic prey revealed conspicuous red hindwings (a colour known to be innately aversive to domestic chicks^49^). Thus chicks received either: (i) Control, (ii) Background-matching/Fast, (iii) Background-matching/Moderate, (iv) Background-matching/Slow, (v) Deimatic/Fast, (vi) Deimatic/Moderate, or (vii) Deimatic/Slow prey.

In the test trial, the prey was always positioned with the bottom edge of the prey parallel to the longer edge of the background images as this allowed the greatest consistency in the approach direction of chicks given the arena layout. The prey was positioned either on the left- or right-hand side of the arena and this positioning was randomly assigned so that half of the chicks received left-hand side positioned prey and the other half received prey on the right-hand side (Fig. 3). At the start of the experimental trial, a chick was put into the experimental arena on the opposite side to the prey, depending on whether the prey was positioned in the right- or left-hand side of the arena. The chick was positioned facing with its head directed away from the prey image and toward the buddy area such that it was not immediately viewing the prey. Upon detecting the prey, chicks invariably walked or ran directly toward it, and when the head of the chick crossed the midpoint of the arena, the experimenter activated the artificial prey using a control keypad. This had the effect of initiating the movement of the forewings (except in stationary control prey where there was no effect). We recorded the time at which the chick first pecked or scratched the artificial prey relative to the time at which it had crossed the midpoint of the arena, using the control keypad. This measure, therefore, represents the deterrent effect of the display. Trials lasted for a maximum of 10 minutes post-display activation, and were video recorded using a JVC Everio GZ-315DEK camcorder.

### Data analysis

We calculated the latency to first contact the prey (the length of time from when the chick crossed the midpoint of the experimental arena until it first attacked (pecked or scratched) the prey), and used this as a measure of how willing birds were to attack displaying prey. Although these data were collected contemporaneously by the experimenter, the interclass correlation coefficient between these measures and those calculated from videos by a naïve observer was extremely high (See supplementary materials). Since these data did not meet the assumptions of parametric tests, we performed a series of planned contrasts using Kruskal-Wallis tests. As there were six degrees of freedom among the seven experimental groups, we tested the following *a priori* predictions based on the ‘startle-first hypothesis’. First, we predicted that birds in the (i) Background-matching/Fast group, (ii) Background-matching/Moderate group, and (iii) Background-matching/Slow group would take longer to attack prey than those in the Control group. That is, movement alone would be sufficient to deter predators. Second, (iv) birds in the Deimatic/Fast group would take significantly longer to attack prey than those in the Background-matching/Fast group; (v) birds in the Deimatic/Moderate group would take significantly longer to attack prey than those in the Background-matching/Moderate group; and (vi) birds in the Deimatic/Slow group would take significantly longer to attack prey than those in the Background-matching/Slow group. That is, movement combined with conspicuous colouration would be more effective than movement alone or no movement at all.

It was not necessary to use Bonferroni or other corrections to control for type I error rate as we carried out a small number of planned comparisons (in line with convention, the number of comparisons did not exceed the degrees of freedom among the experimental groups), testing quite distinct *a priori* predictions^50^. All analyses were carried out using the statistical software R 3.3.2.

### Data availability

The data used in this study are freely available from the data repository Zenodo: 10.5281/zenodo.1212273.

## Electronic supplementary material

Supplementary Materials

Supplementary Video - Background-matching Fast

Supplementary Video - Background-matching Moderate

Supplementary Video - Background-matching Slow

Supplementary Video - Control

Supplementary Video - Deimatic Fast

Supplementary Video - Deimatic Moderate

Supplementary Video - Deimatic Slow
