# Supplementary material for: Testing the feasibility of the startle-first route to deimatism
Source: Sci Rep. 2018 Jul 16;8:10737. doi: 10.1038/s41598-018-28565-w (PMC6048153; doi:10.1038/s41598-018-28565-w)
Supplement: Supplementary file 1 — Supplementary Materials [file 41598_2018_28565_MOESM1_ESM.docx]

**Testing the feasibility of the startle-first route to deimatism**

**Grace G Holmes^1*^, Emeline Delferrière^1^, Candy Rowe^1^, Jolyon Troscianko^2^, John Skelhorn^1^**

^1^Centre for Behaviour & Evolution, Institute of Neuroscience, Newcastle University, Newcastle upon Tyne, UK

^2^Centre for Ecology and Conservation, College of Life & Environmental Sciences, University of Exeter, UK

* Contact GGH: Centre for Behaviour & Evolution, Institute of Neuroscience, Newcastle University, Henry Wellcome Building, Framlington Place. Newcastle upon Tyne, NE2 4HH, UK

Email [g.holmes@ncl.ac.uk](mailto:g.holmes@ncl.ac.uk)

**Supplementary Methods**

1. **Background Images & Training Prey**

*Background images*

Background images were created from 57 monochromatic photographs of tree bark (oak, beech, birch, holly and ash) taken using a Canon 5D MKII with a Nikkor EL 80mm lens at F/22 to ensure a sizable depth of field. These photographs were taken under diffuse light conditions and the images were uploaded to a computer where they were standardized to ensure they had a similar mean luminance and contrast. The images were cropped and scaled to a 1:1 aspect ratio to the monitor’s resolution of 1280 x 800 px using bilinear interpolation (Fig 2A). The pixel values were converted to 32-bit greyscale and log-transformed, which resulted in an approximately normal distribution of pixel luminance values. For each image, a histogram of pixel values with 10,000 bins was analysed. The 1st, 50th and 99th percentile luminance values were calculated, and their bins were modelled using a quadratic function against the desired values for these percentiles to ensure that the median (50^th^ percentile) was half way between the luminance at the upper and lower limits, based on a log scale. The background images all had approximately equal mean and median luminance, similar luminance distributions (i.e. contrast in luminance), and, equal numbers of pixels at their upper and lower extremes.

*Training Prey*

To create the background matching prey, a triangular section of the background image (200px wide by 100px high) was selected from a random location. Prior to threshold modelling a Gaussian gradient was applied to the edges of the prey image which made it less likely that underlying patterns would appear nearer the edge of the prey. This step allowed the avoidance of the creation of salient internal lines in the background-matching prey parallel with the prey’s outline, while also ensuring that no patterns touch the very edges. The prey image was then spilt into 50% pattern and 50% background using thresholding. If the thresholded proportion was not within 1% of the target limits the thresholding process was repeated. Prey images were generated with one of two patterns; dark-on-light or light-on-dark, and each chick received only one of these treatments. The light value of the pattern was equal to the 95th percentile of the background’s luminance, and the dark value was equal to the 5th percentile.

In order to produce the distractive prey, we followed the procedure for producing background-matching prey, but added a single distractive marking to each prey. The distractive markings were created by repeatedly sampling the background image using a thresholding selection tool until a selection area of 50-60 pixels was formed with maximal width and height dimensions not in excess of 20 pixels. This outline of the marking was then filled in with white and randomly placed on the prey image while ensuring that it was not placed within 2 pixels of the prey edge.


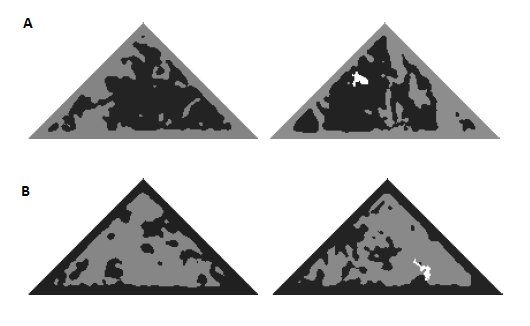


**Figure 2. Examples of training prey.** (A) Dark-on-light prey, (B) Light-on-dark prey. On the left are background-matching and prey on the right have distractive markings.

1. **Camouflage Experiment**

**Test**

On days 10 and 11 post hatch, the chicks participated in an experiment investigating camouflage patterns influence detection^1^. This experiment consisted of two test trials carried out over consecutive days. These trials took the same format as the training trials, but the type and order of prey presented differed among the six experimental groups. The experiment had a 3 x 2 design, whereby in the first 10 presentations in each of the two trials, chicks encountered either all background matching, all disruptive, or 5 of each prey type in a random order; and in the following 10 presentations they encountered either all background matching, or all distractive prey. At the end of this experiment chicks went on to take part in the test trial of the experiment investigating the evolution of deimatism. We ensured that chicks from each of the 6 groups used in the camouflage experiment were counterbalanced across the 7 groups used in our deimatism experiment.

1. **Inter-rater Reliability Analysis**

The design of our experimental system was such that it was impossible for the experimenter to be blind to the experimental aims and manipulations while live scoring the occurrence of attacking behaviour. As the behaviours were so specific, we did not believe that this would influence the data collected. However, in order to ensure that this was indeed the case, we enlisted an independent observer outside of our research group to score the video recordings of test trials. This observer scored i) the point at which the chick’s head crossed the midpoint of the arena (the cue used by the experimenter to activate prey during test trials), and ii) the first time a chick pecked or scratched the prey item. Thus, from this we were able to calculate the latency to attack prey as measured by an independent observer blind to the aims of our experiment. Using the statistical software package IBM SPSS Statistics 23, we compared the latencies scored live by the experimenter and those scored by our independent observer using the Intraclass Correlation Coefficient (ICC). ICCs are measured on a scale of 0 to 1, where 1 represented perfect reliability between observers and 0 represents no reliability. We found that the ICC = 0.896, with 95% Cl (0.829, 0.938). This score provides evidence for the reliability of measurements between our two observers. This result supports the validity of the data presented here.

**References**

1. Troscianko, J., Skelhorn, J. & Stevens, M. Quantifying camouflage how to predict detectability from appearance. *BMC Evolutionary Biology* 17, 7 (2017). doi: 10.1186/s12862-016-0854-2
